# Enhanced Performance of Zn/Br Flow Battery Using *N*-Methyl-*N*-Propylmorpholinium Bromide as Complexing Agent

**DOI:** 10.3390/ijms22179288

**Published:** 2021-08-27

**Authors:** Uxua Jiménez-Blasco, Eduardo Moreno, Maura Cólera, Pilar Díaz-Carrasco, José C. Arrebola, Alvaro Caballero, Julián Morales, Óscar A. Vargas

**Affiliations:** 1 Jofemar Energy, Ctra Marcilla, km. 2, 31350 Peralta, Spain; uxuaj@jofemar.com (U.J.-B.); eduardom@jofemar.com (E.M.); maurac@jofemar.com (M.C.); 2 Departamento Química Inorgánica, Instituto de Investigación en Química Fina y Nanoquímica, Universidad de Córdoba, 14071 Córdoba, Spain or pcarrasco.fcex@ceu.es (P.D.-C.); q92arhaj@uco.es (J.C.A.); iq1mopaj@uco.es (J.M.); 3 Departamento Química y Bioquímica, Universidad CEU San Pablo, 28003 Madrid, Spain; 4 Departamento Didáctica de las Ciencias Experimentales, Facultad de Ciencias de la Educación, Universidad de Córdoba, 14071 Córdoba, Spain; 5 Escuela de Ingeniería Metalúrgica y Ciencia de los Materiales, Universidad Industrial de Santander, Bucaramanga, Santander 680002, Colombia; osavarce@uis.edu.co

**Keywords:** morpholinium-based bromide, complexing agent, catholyte, redox flow batteries

## Abstract

Redox flow batteries (RFB) are one of the most interesting technologies in the field of energy storage, since they allow the decoupling of power and capacity. Zinc–bromine flow batteries (ZBFB) are a type of hybrid RFB, as the capacity depends on the effective area of the negative electrode (anode), on which metallic zinc is deposited during the charging process. Gaseous bromine is generated at the positive electrode (cathode) during the charging process, so the use of bromine complexing agents (BCA) is very important. These BCAs are quaternary amines capable of complexation with bromine and generating an organic phase, immiscible with the aqueous electrolyte. One of the most commonly used BCAs in RFB technology is 4-methylethylmorpholinium bromide (MEM-Br). In this work, an alternative quaternary amine 4-methylpropylmorpholinium bromide (MPM-Br) was studied. MPM-Br was integrated into the electrolyte, and 200 charge–discharge cycles were performed on the resulting ZBFBs. The obtained results were compared with those when MEM-Br was used, and it was observed that the electrolyte with MPM-Br displays a higher resistance in voltage and higher energy efficiency, making it a promising alternative to MEM-Br.

## 1. Introduction

**Figure 1:**
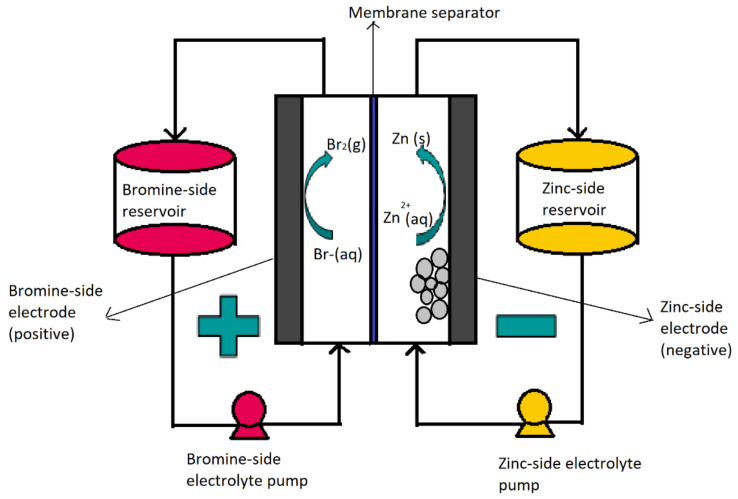
Scheme of a Zn-Br flow battery.

Due to high demand in the field of renewable energy, new energy storage systems have been developed to help balance the generation of these intermittent sources. The Li-ion battery has been one of the technologies with the greatest momentum and demand in the market, attracting enormous interest in the development of advanced functional materials [1,2,3]. Given the high cost of lithium-based materials and their limited availability, the study of other alternative technologies is necessary due to the perpetual rise in the demand for safer rechargeable batteries, such as post-lithium [4] or redox-flow batteries. The zinc/bromine flow battery (ZBFB) is a promising technology, due to its low cost and high energy density [5]. A ZBFB (Figure 1) is a hybrid redox flow battery, in which a large part of the energy is stored in the form of metallic zinc, deposited on the anode. Therefore, the total energy storage capacity of this system depends on the size of the battery (effective electrode area) and the size of the electrolyte storage tanks. For this reason, capacity and power are not totally decoupled in this type of battery [6]. The concept of these batteries first appeared more than 100 years ago, although it was not until the 1970 that Exxon and Gould made the first practical proposals. Bromine–zinc hybrid operating systems can range in energy capacity from 50 to 400 kWh and can supply power for 2–10 h with an energy efficiency of 70% or higher, while their energy density is 65–75 Wh/kg [7,8].

The electrolyte in these batteries is based on zinc bromide, and by pumping this electrolyte through the cells, the oxidation/reduction reactions which allow the battery to charge or discharge take place at the surface of the electrodes in contact with the liquid. During the charging process, the electrolyte is pumped into the cell, where bromine gas is formed in the cathodic region by oxidation of bromide, while in the anodic region, the Zn^2+^ is reduced to metallic zinc and deposited on the surface of the electrode, as shown in Figure 1 [9,10,11].

Bromine has limited solubility in water, and so a bromine complexing agent (BCA) or quaternary amine is added to the electrolyte, in order to capture the bromine formed during the charging process and to prevent its evaporation (boiling point of bromine = 58 °C). As the bromine–BCA complex is formed, a high-density immiscible liquid is formed, which sinks to the bottom of the positive electrolyte (catholyte) tank. During the discharge process, the immiscible liquid must be mixed with the rest of the catholyte in order to transport and release the bromine molecules on the surface of the positive electrode. In addition, the metallic zinc previously formed at the anode is oxidised to Zn^2+^ ions, and dissolves into the anolyte (negative electrolyte), releasing two electrons which are transported by the external circuit. The electrons return to the cathode and reduce the bromine molecules to bromide ions, which are soluble in the catholyte. The chemical process used to generate the electric current increases the concentration of zinc and bromide ions in both electrolytes [12].

In ZBFB-type batteries, the corrosion and toxicity which arise due to free bromine are the main problems to be solved. To mitigate these problems, it is essential to use a BCA capable of creating an organic liquid phase in the aqueous electrolyte by trapping free bromine formed during the charging stage (Table 1) [13]. One of the most important requirements is optimising the kinetics of the complexation mechanism between the BCA and free bromine. If this proceeds too slowly, the free bromine that forms on the surface of the electrode may escape from the cell, or diffuse to the other electrode of the cell, causing complete self-discharge. Consequently, the reverse reaction, in which bromine is reduced to bromide, would not occur, preventing discharge of the battery. The organic liquid phase, formed by free bromine and the BCA, remains at the bottom of the electrolyte reservoir, immiscible in the aqueous liquid phase [14].

In this manner, different quaternary amines have been used as BCAs in commercial batteries, particularly complexes such as methylethylmorpholinium bromide (MEM) or methylethylpyrrolidinium bromide (MEP), or their mixture [5,13,19,20,21]. Additional quaternary ammonium complexes which capture the electro-generated bromine but retain it in the aqueous phase have also been examined. Scheneider et al. investigated five alternative BCA candidates (1-ethyl-1-methylpiperidinium bromide, 1-(2-hydroxyethyl)-pyridinium bromide, 1-ethylpyridinium bromide, 1-ethyl-3-methylimidazolium bromide and 1-(2-hydroxyethyl)-3-methylimidazolium bromide), demonstrating an improvement in electrochemical performance compared to the conventional BCA (MEM) [12]. Bryans et al. analysed three compounds (1-(carboxymethyl)pyridin-1-ium, 1-(2-carboxymethyl)-1-methylmorpholin-1-ium and 1-(2-carboxymethyl)-1-methylpyrrolidin-1-ium), and all were successfully used to reduce the volume of the immiscible phase formed upon complexation with the polybromide [18].

**Figure 2:**
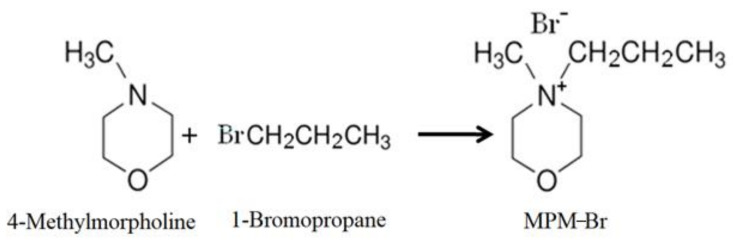
Second degree (SN2) nucleophilic substitution reaction forming MPM-Br salt.

The use of a suitable complexing agent is of high importance in the design of this type of battery, as it is a fundamental part of its proper function. In this work, we investigated the physicochemical and electrochemical properties of a new BCA, namely *N*-methyl-*N*-propylmorpholinium bromide [MPM-Br]. The complexing agent was prepared via a second order nucleophilic substitution reaction, in which 4-methylmorpholine is the base molecule, 1-bromopropane acts as the adduct, and the solvent is acetonitrile (a reaction scheme is shown in Figure 2). The physicochemical properties were obtained as a function of composition, structure, molecular weight and thermal response, and the electrochemical properties were obtained from a ZBFB designed by Jofemar S.A. The MPM-Br complexing agent prepared here by a simple and rapid process demonstrates improved performance in the redox flow battery, compared to the conventional MEM complexing agent.

## 2. Results and Discussion

### 2.1. Synthesis of MPM-Br Salt

Table 2 summarises the results obtained with variations in the synthetic procedure for MPM-Br salt. According to these results, it can be concluded that acetone is not a suitable option to replace acetonitrile as the solvent in this reaction, and reaction without any solvent is also unfeasible, due to very low yields. However, diminishing the amount of acetonitrile by half is a potential option, an approach which gives the maximum product yield achieved in this work (90%), a slight increase compared to the yield of the synthesis using the full amount of solvent (85%). The adduct, 1-bromopropane, must be added in excess in order to balance losses due to its evaporation. In addition, it is necessary to reduce its vapour in the reaction vessel, as it forms explosive mixtures with air. Despite this, it is not recommended to use less than an excess amount of the adduct, as there is a direct relationship between excess of adduct and yield of product (Table 2). For this reason, the use of both reflux and nitrogen bubbling inside the vessel is unavoidable.

**Figure 3:**
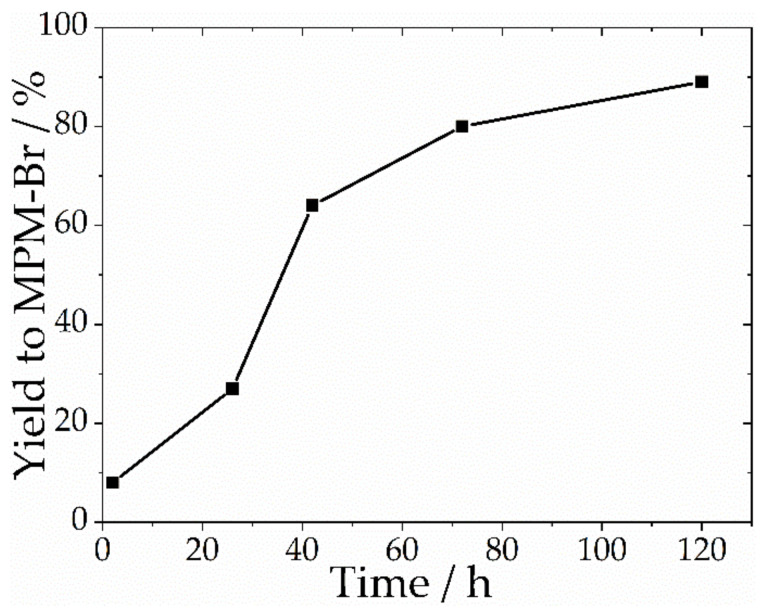
Kinetics of the reaction at ambient temperature for MPM-Br synthesis.

Temperature can be used as a tool for improving reaction kinetics, but is also responsible for adduct volatilisation; however, a decrease in temperature from 70 °C to ambient temperature (25 °C) increased the crystallisation time (Table 2). Figure 3 shows a kinetics study for the reaction of 1-methylmorpholine and a 50% excess of 1-bromopropane at ambient temperature. After 5 h agitation, the reactants were allowed to settle and crystallise over different lengths of time. Over the same crystallisation time as for the reactions performed at higher temperatures, the product yield barely reached 20%. To achieve similar yields as the reactions at higher temperatures, it was necessary to extend the crystallisation time to 120 h, 10 times the time required for reactions at higher temperatures.

To the best of our knowledge, the synthesis of MPM-Br has not been reported in detail. The sole report is from Zawadski et al. [22], which describes obtaining ca. 94% yield using a 10% excess of 1-bromopropane. However, no other reaction conditions were described, such as reaction temperature, which solvent was used, or whether or not oxygen was displaced; for this reason, it is difficult to draw a comparison between synthetic protocols. It may be assumed that a similar procedure to those reported for the synthesis of MEM-Br in several reports [15,20,23,24], as was used initially in this work; these procedures are summarised in Table 3, using 1-methylmorpholine and bromoethane as reactants in acetonitrile as solvent, unless otherwise stated. Product yields from the four cited reports are identical (90%), despite some differences in reaction conditions. The main difference between this work and the literature is in terms of yield; yield is lower in this work, likely due to the decrease in solvent amount, which may lead to steric hindrance. Despite this, it was decided to conduct the reaction forming MPM-Br using a lower amount of acetonitrile, but using a higher excess of 1-bromopropane, with an additional crystallisation step. The synthesis reported here for MPM-Br under conditions of ambient temperature shows considerable optimisation, in comparison to those previously reported (Table 3).

### 2.2. Physicochemical Properties of MPM-Br

**Figure 4:**
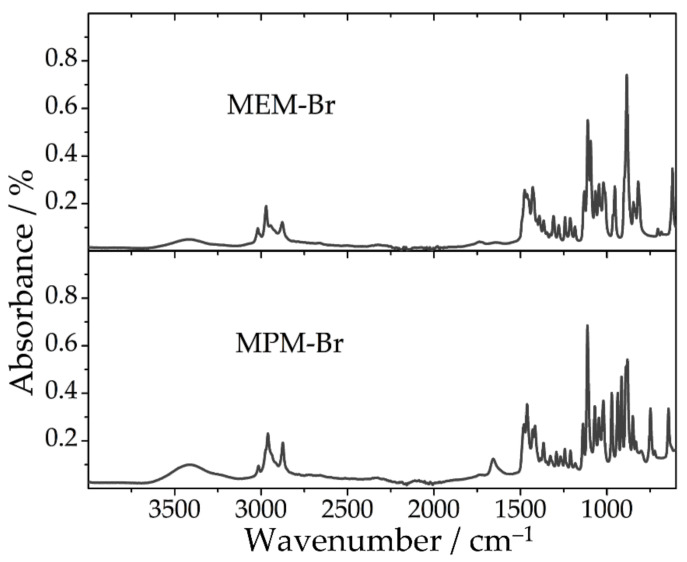
FTIR spectra of MPM-Br and MEM-Br.

The FTIR spectra of MPM-Br and MEM-Br are shown in Figure 4, showing several peaks highlighting the differences between these two compounds. According to Kautek et al. [25], the region from 1300 to 1080 cm^−1^, with the dominant peak at 1130 cm^−1^, contains characteristic signals of MEM-Br. In the spectra in this work, there are some intense peaks in this region and the dominant peak is centered near 1110 cm^−1^. However, this region is not considered characteristic of MEM-Br, as MPM-Br also exhibits some important peaks in this range. The key difference is that MEM-Br presents three peaks (at 1134, 1110 and 1094 cm^−1^) while MPM-Br presents just two peaks (at 1140 and 1113 cm^−1^), likely corresponding to those ascribed to C–N and C–O in the morpholinium group [26]. No reports have been found in the literature regarding the infrared spectra of MPM-Br. According to correlation charts [26], the more intense peaks are assigned as in Table 4.

**Figure 5:**
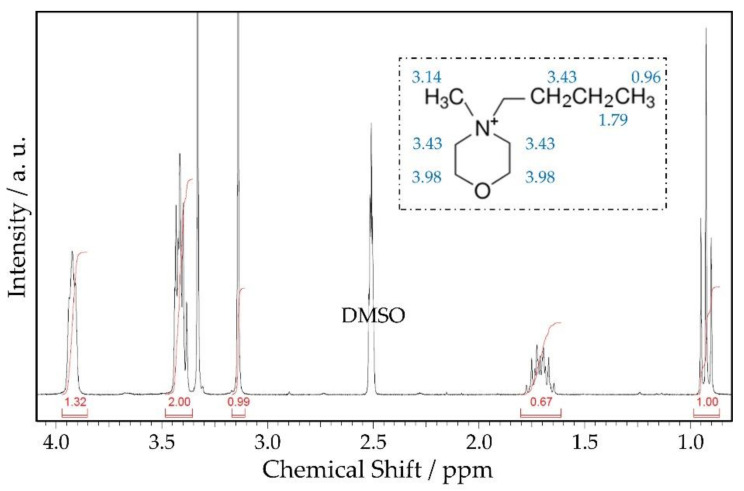
^1^H-NMR spectrum of MPM-Br. In the inset, the molecule of MPM-Br and the assignation of the ^1^H-NMR peaks.

Figure 5 shows the ^1^H-NMR spectrum for the synthesised salt; the inset in this figure, as well as Table 5, shows the assignation of peaks to each functional group. The signal at 2.5 ppm corresponds to the deuterated DMSO solvent.

The ^1^H-NMR spectroscopy results presented are partially in agreement with those obtained by Zawadski et al. [27]; although, as shown in Table 5, there are slight differences in chemical shift for all peaks. These differences may be attributed to the solvent used in the analysis; here, DMSO was used, instead of D_2_O as in the cited work.

HPLC-MS was used in order to determine the molecular weight of the synthesised salt. The mass fraction analysis shows a molecular weight of 144 g mol^−1^, corresponding to the organic cation C_8_H_18_NO^+^, part of the *N*-propyl-*N*-methylmorpholinium bromide salt.

**Figure 6:**
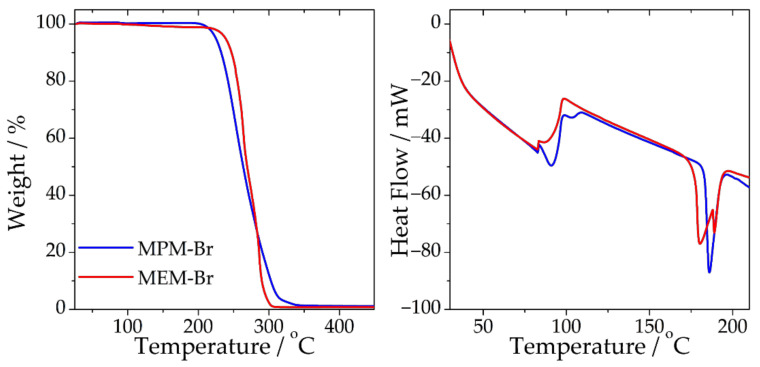
Thermogravimetric analysis (**left**) and differential scanning calorimetric analysis (**right**) of the synthesised complexing salts MPM-Br and MEM-Br.

Figure 6 shows thermal properties of the salt. The salt’s melting point, as indicated by the sharp exothermic peak, is approximately 185 °C. Zawadski et al. [22] reported a lower melting point, near 181 °C, and also a phase transition temperature near 68 °C; however, the nature of this transition is not explained. The DSC data in the report from Zawadski et al. shows a sharp endothermic peak at 68 °C, whereas ours exhibit a wide peak at approximately 90 °C, which is associated with water release. For the decomposition temperature, as shown from TGA data, the results are in agreement with Zawadski et al., showing a decomposition starting at 200 °C in both cases. Melting point does not follow a trend with the length of the substituting branch in *N*-alkyl-*N*-methylmorpholinium bromides, as reported by Zawadski et al. [22] and Cha et al. [15] (methyl: 189 °C, butyl: 210 °C, pentyl: 194 °C, octyl: 155 °C, dodecyl: 141 °C), resulting in difficulty in predicting the melting point of the propyl-substituted compound.

### 2.3. Physicochemical Properties of Electrolytes

Table 6 shows a comparison between the physicochemical properties of electrolytes formulated with MPM-Br and MEM-Br as bromine complexing agents. No remarkable difference was observed between electrolytes in terms of conductivity, pH and density. Nevertheless, a large difference in viscosity was observed, a property directly affecting fluid mechanics, which is paramount in studying these types of batteries. The conductivity results are in good agreement with Lancry et al. [28], who did not find significant differences between conductivities of electrolytes formulated with *N*-alkyl-*N*-methylmorpholinium bromides substituted with ethyl, *n*-propyl, *iso*-propyl, *n*-butyl and iso-butyl branches. The physicochemical properties of the electrolyte (especially conductivity and pH) hardly change if the electrolyte is exposed to air for a long time.

### 2.4. Electrochemical Characterisation

#### 2.4.1. Design and Assembly of the Cell Prototype

**Figure 7:**
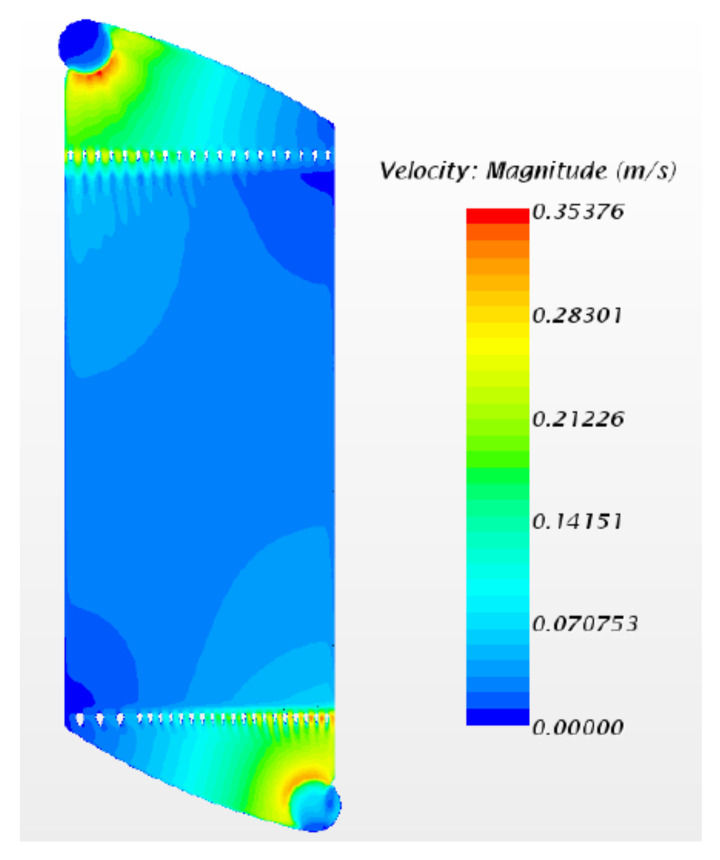
Simulation of the flow velocity in cell model 1, from *Ansys* simulation and design software.

**Figure 8:**
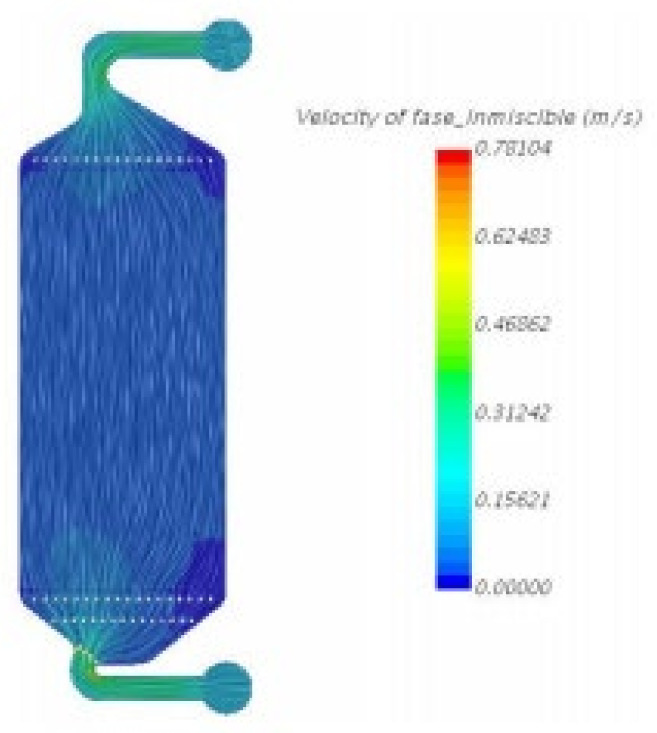
Simulation of the flow velocity in cell model 2, from *Ansys* simulation and design software.

With *Ansys* simulation and design software, the cell design was optimised for homogeneous electrolyte flow. In Figure 7, it is observed that the velocity of the electrolyte flow does not change when it passes though the electrode. The higher flow velocity was observed at the entry and exit points of the electrolyte in the cell. In Figure 8, other cell designs are shown, where the flow rate is higher than in the design in Figure 7. The flow rate is of high importance, as if it is not sufficiently high, the immiscible phase may deposit and clog the system. When drawing comparison between the designs of Figure 7 and Figure 8, it may be said that the optimised cell design is that observed in Figure 8.

In addition to obtaining a better result for the flow rate, the design shown in Figure 8 also solves the issue created by shunt currents, due to the increase in the liquid path.

#### 2.4.2. Electrochemical Performance

**Figure 9:**
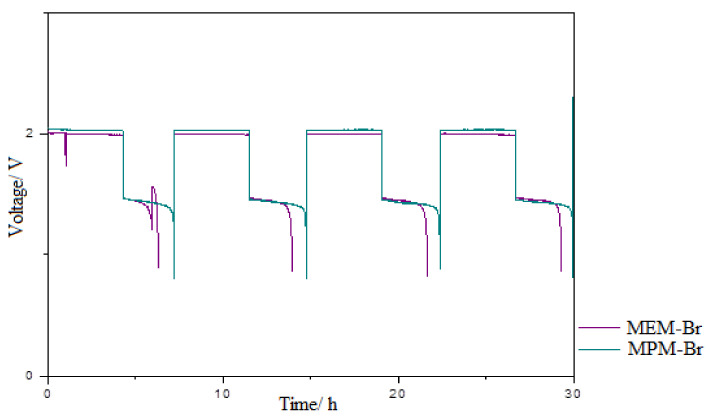
Profile of charge–discharge cycles for MPM-Br and MEM-Br based ZBFBs at 1 M of BCA in each electrolyte where ZnBr_2_ concentration is 2 M and 20 mA/cm^2^ current density.

Galvanostatic charge–discharge cycles controlling cell voltage and temperature were achieved. Figure 9 shows a charge–discharge profile in which the voltages of the cells are observed over four cycles. As reflected in the graph, the charge voltage was 1.9 V and the discharge began at 1.6 V for the MPM-Br-based cell. The charge voltage remained relatively constant, although there a small upward slope was apparent, as the voltage of the cell at the end of charging is higher than at the beginning of charging. During discharge, the profile is practically flat, focusing the discharge voltage between 1.4 and 1.5 V. The voltage drop to 0.8 V occurs during a very short time interval.

The battery containing MEM-Br complexing agent shows a higher cell voltage during the charging and lower cell voltage during discharging. This occurs due to the higher resistance of the battery containing MEM-Br vs. MPM-Br.

**Figure 10:**
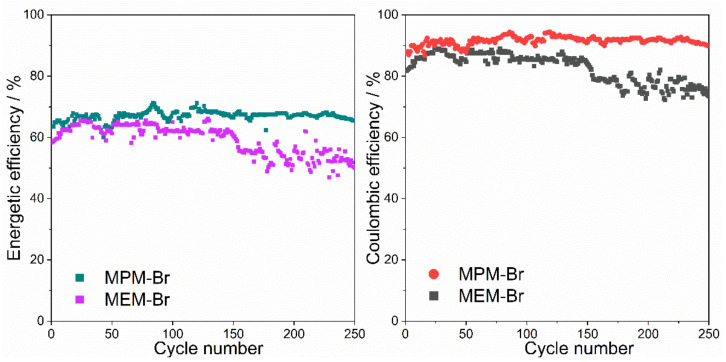
Energy (**left**) and coulombic (**right**) efficiencies of batteries for MPM-Br and MEM-Br based ZBFBs at 1 M of BCA in each electrolyte where ZnBr_2_ concentration is 2 M and 20 mA/cm^2^ current density.

The energy and coulombic efficiencies of the two batteries are compared in Figure 10. In the graph, it can be seen that the energetic efficiency of the battery containing MEM-Br starts at 65% and decreases during cycling, until reaching 60% after 200 cycles. In the case of the battery containing MPM-Br, the initial energy efficiency is 70%, and the efficiency remains at this value throughout cycling. Therefore, the percentage improvement in performance of MPM-Br compared to the control group is around 10% after 200 cycles. The coulombic efficiency for the MPM-Br electrolyte-based cell presents higher and more stable values (around 90% efficiency) than the battery containing MEM-Br, throughout the prolonged cycling.

The results are, therefore, promising for the MPM-Br complexing agent, compared to the standard MEM-Br, in terms of both energy performance and stability.

**Figure 11:**
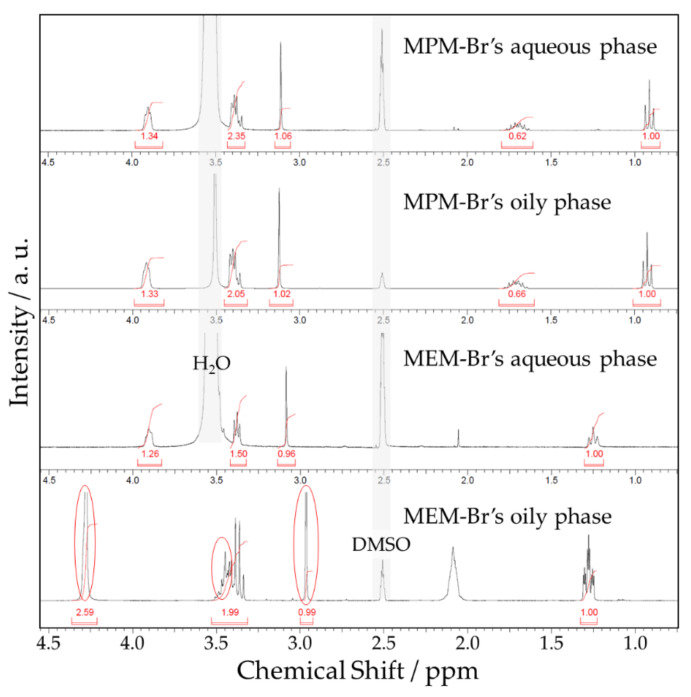
^1^H-NMR spectra of electrolytes after 250 cycles of charge/discharge.

In order to gain better knowledge about the reasons for the difference in electrochemical performance, two analyses were performed. ^1^H-NMR spectra of the cycled electrolytes were acquired in order to compare the structures of the complexing salts before and after electrochemical activity. During charging, bromine is formed from bromide, and is supposed to be captured by the complexing agent; the complex is an oily and heavy substance which precipitates, forming a two-phase system in the charged electrolyte. Figure 11 shows the ^1^H-NMR spectra of both phases; for both electrolytes, the spectra from the aqueous phases contain the entire corresponding salt profile, indicating that the salt remains unchanged in the diluted form. However, the spectra of the oily phases exhibit remarkable differences compared to the aqueous phases. For the MPM-Br electrolyte oily phase, the peak profile is identical to that of the original salt, indicating that the structure of the complexing salt is maintained even after the electrochemical activity. In contrast, the spectrum of the MEM-Br oily phase exhibits a group of new peaks not present in the original salt. Though unconfirmed, we hypothesise that this may be due to a Hoffman’s degradation promoted by the excess bromine and side electrochemical reactions, in which the hetero-cycle opens of the molecule opens, and the double bonds formed displace the chemical shift to higher ppm values.

Iodometric titration was performed to measure the amount of free bromine in the aqueous electrolyte, i.e., bromine which the complexing agent was not able to complexate, providing information on the ability of the salt to capture bromine. The higher the free bromine content in the electrolyte, the worse the ability of the salt to capture bromine, and the lower the efficiency of the cell. Lancry et al. [28] reported a five-fold lower amount of free bromine in electrolytes with *n*-propyl and iso-propyl substituted *N*-alkyl-*N*-methylmorpholinium bromide than in electrolytes with the ethyl-substituted salt; the amount of free bromine was lowered as the branch length increased. In this work, the MEM-Br based electrolyte had a free bromine content of 2.92 mmol/g, while the MPM-Br based electrolyte had 3.6 times lower bromine content (0.82 mmol/g).

The different N-alkylated groups on N-methylmorpholinium bromide yield interesting behaviour in terms of performance as a bromine complexing agent. E. Lancy et al. [28] addressed this topic by studying the effect of the length of the N-alkylated substitute group. They reported two key parameters that will determine the performance of BCA in redox-flow battery electrolyte. On the one hand, they determined that the specific conductivity of the electrolyte decreases with increasing chain length, from ethyl to propyl to N-alkylated *N*-methylpyrrolidinium. On the other hand, the free bromine concentration in the electrolyte aqueous phase, i.e., the amount of bromine that is not complexed by the BCA was also studied, since it is one of the most important parameters for the selection of BCA. Like the data obtained in our work, they reported that the free aqueous bromine concentration measured for the MEP-Br derivatives decreased with substitute lengthening. Reduction of bromine concentration in the electrolyte aqueous phase will be reflected in improved coulombic efficiency of the cell. This data can explain the better result of the MPM-Br with respect to the MEM-Br as BCA, given that the optimal free bromine concentration is the lowest possible, as long as the availability of bromine for the electrochemical process is not becoming a constraint. Results regarding the integrity of the complexing salt and free bromine indicate that the MPM-Br complexing agent is more efficient at capturing bromine, hence its superior performance during long cycling experiments. Additionally, further work is required in order to corroborate this better performance under higher current densities.

## 3. Materials and Methods

### 3.1. Electrolyte Preparation

For the synthesis of the complexing agent, the reaction temperature was set to 70 °C; as the boiling points of bromopropane and acetonitrile are both close to this temperature, it was necessary to use a condenser to reflux the vapours. As bromopropane and oxygen are able to form an explosive mixture, a nitrogen flow (50 mL/min) was introduced to the reaction vessel in order to displace the oxygen atmosphere. The mixture was allowed to react over 5 h at the reaction temperature, after which the system was allowed to naturally cool to ambient temperature, and then a crystallisation stage between 2 and 120 h was necessary to obtain the salt. In order to purify the product, a rotavapor system was used at 70 °C under vacuum. The unreacted components and remaining solvent were both extracted in this step. The adduct was added in 14–60% excess. The solvent volume was 100 mL, except in two cases where solvent was either not added or added in half this amount. Two additional variations to the reaction were later introduced. First, acetone was used instead of acetonitrile as the solvent, in which case the temperature was set to 50 °C, taking into account the lower boiling point of acetone. The second variation performing the reaction at ambient temperature instead of 70 °C; in this case, it was not necessary to use the condenser or nitrogen flow. For this synthesis, 4-methylmorpholine (98%), 1-bromopropane (99%) and acetonitrile (99%) were purchased from Alpha Chemika (Mumbai, India), and acetone (99.5%) from Panreac (Castellar del Vallès, Spain). *N*-methyl-*N*-ethylmorpholinium bromide (MEM-Br) was also synthesised and characterised, for comparison.

**Figure 12:**
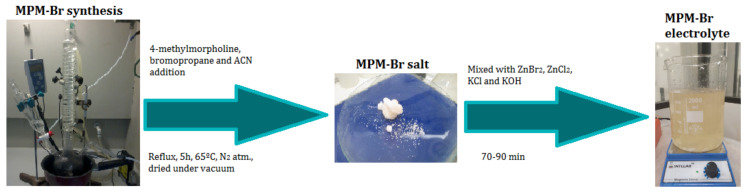
Scheme of MPM-Br-based electrolyte preparation.

The electrolyte is arguably the most important component in flow batteries. Unlike traditional batteries, in these systems electrolyte is an active component, i.e., the component which undergoes redox reactions. In this study, the electrolyte was formulated with commercial-grade reactants (Sigma-Aldrich, St. Louis, MO, USA), with the exception of the complexing agent which was synthesised in-house. The components of the electrolyte and their concentrations are ZnBr_2_ (2 M, 98%) as the active salt, ZnCl_2_ (1 M, 98%) as an additional Zn^2+^ source, KCl (2 M, 99%) as a conductivity enhancer, KOH (0.05 M, 85%) as pH corrector and MPM-Br (1 M) as BCA. All components were added stepwise, as the mixture is exothermic, into distilled water under continuous agitation (Figure 12).

### 3.2. Characterisation of the Electrolyte

For complexing agent characterisation, the structure, molecular weight, thermal properties and purity were elucidated using various techniques. Infrared spectroscopy with attenuated total reflectance (FTIR-ATR) was used to identify functional groups in the salt; samples were analysed in the range 3800–800 cm^−1^ using a Cary 360 instrument (Agilent). Proton nuclear magnetic resonance (^1^H-NMR) was used to identify the structure of MPM-Br, using deuterated dimethyl sulfoxide (DMSO) as solvent in a Bruker spectrometer Avance III WB operated at 600 MHz. High performance liquid chromatography coupled with mass spectrometry (HPLC-MS) was used to find the molecular weight of the synthesised salt. Thermogravimetric analysis (TGA) and differential scanning calorimetry (DSC) were performed in order to elucidate thermal characteristics including melting point and decomposition temperature, and was performed using a Mettler Toledo TGA/DSC-1, under nitrogen atmosphere at a heating rate of 0.3 °C/min.

Once the MPM-Br salt was synthesised, the purest obtained salts were used for formulation of the electrolyte, as previously mentioned. Several characterisation techniques were used in order to calculate physicochemical properties of the prepared electrolytes and, also, to completely characterise the synthesised MPM-Br salt. The electrolyte properties measured were pH and conductivity, using a pH-meter (DAGATRON AG217); viscosity, using a Cannon-Fenske viscometer. Post-cycling characterisation was performed on electrolytes formulated with MPM-Br and MEM-Br, in order to shed light on the differences in electrochemical performance. ^1^H-NMR and iodometric titration were used to characterise the complexing agent integrity and the amount of free bromine, respectively.

### 3.3. Electrochemical Characterisation

To test the behaviour of the complexing agent MPM-Br in a realistic setting, charge and discharge cycles were performed in a ZBFB.

#### 3.3.1. Design and Assembly of the Cell Prototype

For realistic testing of the complexing agent, a ZBFB was designed. Several studies were carried out to obtain the optimal cell design, using *Ansys* simulation and design software [29]. Ideally there was a homogeneous flow, i.e., no areas where the electrolyte remains stagnant, no swirl formation and constant flow speed.

**Figure 13:**
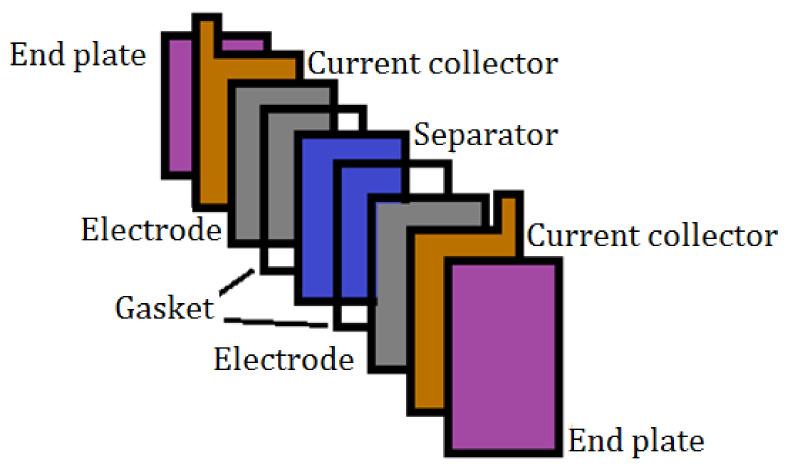
Diagram of the cell prototype assembly.

The following components were used to assemble the cell, once the design was decided upon: composite electrodes (mixture of graphite and binder polymer), a separating membrane (polypropilene microporous monolayer separator, 25 µm, Celgard 3401), gaskets, current collectors and end plates (Figure 13) [30].

The assembly of the hydraulic circuit consists of pumps (which allow the electrolyte to flow), electrolyte reservoirs, pipes and connectors. On the cathodic side, a magnetic stirring rod was placed inside the electrolyte reservoir and a stirrer was placed under the reservoir. Due to this agitation, when the electrolyte is charged, the two immiscible phases present in the battery’s charged state can be mixed.

#### 3.3.2. Electrochemical Performance

For cell operation, it was necessary to introduce electrolyte (600 mL) into each electrolyte reservoir. The charge–discharge cycles were performed at constant current, stopping the charge by time and the discharge by tension (0.8 V). These types of batteries are typically charged and discharged using currents densities ranging from 15 to 30 mA/cm^2^. The capacity of the battery is related to the amount of zinc that can be deposited at the negative electrode, in the range of 60–150 mAh/cm^2^. The amount of electrolyte that can react at the electrodes depends on the battery’s application. When battery efficiency is the most important, approximately 50–70% of electrolyte reacts in order to maximise the efficiency [24]. In this work, the total capacity of the battery was 125 mAh/cm^2^, the fraction of reacted electrolyte was 60%, and the current density was fixed at 20 mA/cm^2^.

Temperature is of high importance for the performance of ZBFBs, and operating temperatures between 20 and 40 °C are recommended [7]. At temperatures lower than 20 °C, the organic phase may solidify; if the temperature is higher than 40 °C, the bromine may not complexate with the complexing agent. If this issue occurs, the efficiency of the cell is lowered. In this study, the temperature of the battery tanks was controlled between the recommended operational range.

## 4. Conclusions

The synthesis and characterisation of the MPM-Br complexing agent was performed. From the use of different techniques, it was possible to confirm that, with the proposed synthesis, the desired salt was obtained as product. The electrolyte was prepared using MPM-Br as a complexing agent, and characterised using a range of techniques in order to verify whether or not its use in ZBFBs was viable. Physicochemical properties of the electrolyte containing MPM-Br were similar to those of the electrolyte with the standard complexing agent MEM-Br, motivating the electrolyte’s further use in a flow battery. The results obtained after cycling of the battery were compared with those of a battery containing MEM-Br as complexing agent. The battery containing MEM-Br in the electrolyte showed higher resistance and lower efficiency compared to the battery containing MPM-Br. The reason for this difference in performance may be the lower bromine-capturing ability of MEM-Br, and the degradation of the same salt during long cycling periods.

## Figures and Tables

**Table 1:** Preparation conditions for the most common BCAs.

BCA	Structure	Precursors	Synthesis Conditions				Dried Under Vacuum	[C]	Ref.
			Tª (°C)	Time	Reflux	Atmosphere			
*N*-ethyl-*N*-methylmorpholinium bromide	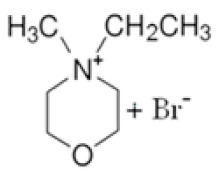	4-methylmorpholine, bromoethane, acetonitrile	65	5 h	Yes	N_2_	Yes	1–3 M	[15,16]
1-ethyl-1-methylpyrrolidinium bromide	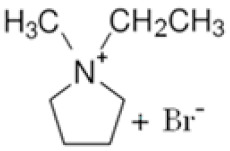	*N*-methylpyrrolidine, bromoethane, acetonitrile	RT	24 h	No	No	Yes	1–3 M	[5,17]
*N*-propyl-*N*-methylmorpholinium bromide	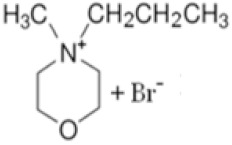	4-methylmorpholine, bromopropane, acetonitrile	65	5 h	Yes	N_2_	Yes	1–3 M	[15]
1-(carboxymethyl) pyridine-1-ium bromide	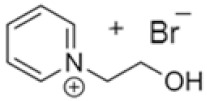	Pyridine, 2-bromoacetic acid, ethyl acetate	RT	4 h	Yes	No	No	1–3 M	[5,18]
1-(2-carboximethyl)-1-methylmorpholinium bromide	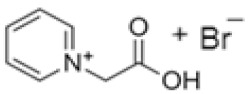	4-methylmorpholine, 3-chloropropanoic acid	RT	4 h	No	No	Yes	1–3 M	[5,18]
1-(2-carboximethyl)-1-methylpyrrolidinium bromide	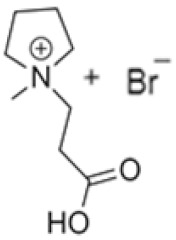	N-methylpyrrolidine, 3-chloropropanoic acid	RT	4 h	No	No	Yes	1–3 M	[5,18]

**Table 2:** Summary of the synthesis of MPM-Br.

Temp. (°C)	Solvent (mL/g Reactants)	Adduct Excess (%)	N_2_	Reflux	Crystallisation (h)	Yield (%)
70	1.41	14	Yes	Yes	12	68
70	1.11	60	Yes	Yes	12	85
70	0	60	Yes	Yes	12	19
50	1.11 *	60	Yes	Yes	12	10
70	0.55	60	Yes	Yes	2	90
25	0.59	50	No	No	120	89

* Acetone was used as the solvent instead of acetonitrile.

**Table 3:** Summary of the synthesis of MEM-Br and comparison with literature.

Temp. (°C)	Solvent (mL/g Reactants)	Adduct Excess (%)	N_2_	Time (h)	Yield (%)	Reference
N.R.	N.R.*	300	N.R.	N.R.	90	[17]
60	2.74	35	Yes	5	90	[25]
N.R.	3.22	0	Yes	5	90	[26]
70	3.22	0	Yes	5	90	[27]
70	1.61	0	Yes	5	78	This work

N.R.: not reported. *Acetone was used as the solvent instead of acetonitrile.

**Table 4:** Functional groups according to correlation charts.

Functional Group	Frequency Range (cm^−1^)	Frequency in MPM-Br Spectra * (cm^−1^)
C–H	3010–3100 (aromatic) 2850–2970 (alkane) 1340–1470 (alkane) 690–900 (aromatic)	3016 2960, 2874 1462 974, 937, 914, 881, 851, 748
C–N	1180–1360	1140, 1113
C–O	1050–1300	1140, 1113

* For the most intense peaks, underlined frequencies are those peaks that do not appear in MEM-Br spectra.

**Table 5:** Chemical shift of protons with the assigned functional groups.

Shift (ppm)	Functional Group		Shift (ppm) Ref. [20]
0.96	-CH_3_	Outer methyl group of the propyl branch	1.03
1.79	-CH_2_-	Inner methyl group of the propyl branch	1.85
3.14	-CH_3_	Methyl branch bound to N	3.22
3.43	-CH_2_-	Three methyl groups bound to N: one from the propyl branch and two from inside the aromatic ring	3.51
3.98	-CH_2_-	Two methyl groups inside the aromatic ring bound to O	4.08

**Table 6:** Physicochemical properties of electrolytes containing different complexing salts.

Complexing Salt	Conductivity (mS/cm)	pH	Density (g/mL)	Viscosity
MPM-Br	94.6	4.4	1.67	3.58
MEM-Br	96.4	4.6	1.53	2.73

## Data Availability

The data presented in this study are available on request from the corresponding author.
